# Building a Human Digital Twin (HDTwin) Using Large Language Models for Cognitive Diagnosis: Algorithm Development and Validation

**DOI:** 10.2196/63866

**Published:** 2024-12-23

**Authors:** Gina Sprint, Maureen Schmitter-Edgecombe, Diane Cook

**Affiliations:** 1 Department of Computer Science Gonzaga University Spokane, WA United States; 2 School of Electrical Engineering and Computer Science Washington State University Pullman, WA United States

**Keywords:** human digital twin, cognitive health, cognitive diagnosis, large language models, artificial intelligence, machine learning, digital behavior marker, interview marker, health information, chatbot, digital twin, smartwatch

## Abstract

**Background:**

Human digital twins have the potential to change the practice of personalizing cognitive health diagnosis because these systems can integrate multiple sources of health information and influence into a unified model. Cognitive health is multifaceted, yet researchers and clinical professionals struggle to align diverse sources of information into a single model.

**Objective:**

This study aims to introduce a method called HDTwin, for unifying heterogeneous data using large language models. HDTwin is designed to predict cognitive diagnoses and offer explanations for its inferences.

**Methods:**

HDTwin integrates cognitive health data from multiple sources, including demographic, behavioral, ecological momentary assessment, *n*-back test, speech, and baseline experimenter testing session markers. Data are converted into text prompts for a large language model. The system then combines these inputs with relevant external knowledge from scientific literature to construct a predictive model. The model’s performance is validated using data from 3 studies involving 124 participants, comparing its diagnostic accuracy with baseline machine learning classifiers.

**Results:**

HDTwin achieves a peak accuracy of 0.81 based on the automated selection of markers, significantly outperforming baseline classifiers. On average, HDTwin yielded accuracy=0.77, precision=0.88, recall=0.63, and Matthews correlation coefficient=0.57. In comparison, the baseline classifiers yielded average accuracy=0.65, precision=0.86, recall=0.35, and Matthews correlation coefficient=0.36. The experiments also reveal that HDTwin yields superior predictive accuracy when information sources are fused compared to single sources. HDTwin’s chatbot interface provides interactive dialogues, aiding in diagnosis interpretation and allowing further exploration of patient data.

**Conclusions:**

HDTwin integrates diverse cognitive health data, enhancing the accuracy and explainability of cognitive diagnoses. This approach outperforms traditional models and provides an interface for navigating patient information. The approach shows promise for improving early detection and intervention strategies in cognitive health.

## Introduction

### Background

Mild cognitive impairment (MCI) is a transition state between healthy aging and dementia. Digital health technologies can enhance early detection and improve the ecological validity of traditional MCI diagnostic assessments. Current technology-assisted approaches often focus on a small set of data sources, such as speech and text [1,2], mobile tests [3,4], self-reported in-the-moment states [5], and digital behavior markers [6-9]. Each contributes valuable insights, but they are fragmented.

We propose the construction of a human digital twin that uses these diverse pieces of digital information to form a more comprehensive model of an individual. This digital twin integrates data from multiple sources, recorded at different times and in real-world settings. The result offers a holistic view that enhances early diagnosis of cognitive impairment and facilitates timely interventions to slow progression, in line with the quest for precision health.

Designing digital twins faces the challenges associated with merging data that differ in acquisition times, devices, formats, and fidelity. Clinicians also need help navigating these types of information with traditional dashboards [10]. To overcome these challenges, we propose a system called HDTwin that uses large language models (LLMs) to create a cohesive digital twin from heterogeneous data sources. In this paper, we detail the design of HDTwin and evaluate the system in the context of automating cognitive health diagnosis for 124 participants from 3 studies. Specifically, we highlight the system’s ability to perform information retrieval, data fusion, and inference explanations. We demonstrate that HDTwin successfully integrates traditional machine learning models, numeric reasoning resources, scientific literature, audio recordings, and ecological momentary assessment (EMA) responses to generate diagnosis predictions that are comparable or superior to traditional ensemble classifiers. We further investigate the ability of HDTwin to process information and articulate clear diagnostic explanations interactively, providing a bidirectional flow of information between a clinician and the computational model.

### Related Work

Recent advances in language models have tremendously impacted health question answering and information retrieval. These prior works focus on leveraging specialized corpora for enhanced performance. Language models are trained on biomedical texts to refine their capabilities in summarizing documents and answering complex health-related questions [11,12]. Models have been further enhanced by efforts like KeBioSum [13], which integrates medical knowledge into model training to improve response accuracy.

Language models also support health prediction. As an example, AD-BERT [14] processes electronic health record notes with pretrained models to forecast a patient’s progression from MCI to Alzheimer disease. Research by Asgari et al [15] leverages text markers from recorded speech to predict MCI. Jiang et al [16] train an LLM on medical language to predict hospital readmission, and Kim et al [17] evaluate prompting strategies for LLMs on a variety of health prediction tasks. Because LLMs can inherently predict future states, Xue and Salim [18] explore pretrained LLMs to predict future temperature, electricity consumption, and movement trajectories. Similarly, Sprint et al [19] demonstrated that LLMs could anticipate future health states based on past EMA reports and sensor-based behavior data.

Recently, researchers have extended LLM capabilities to interpret nontextual data inputs. Yu et al [20] direct LLMs to diagnose sleep apnea and cardiac conditions by leveraging large databases. Jin et al [21] convert time-series data into text to forecast electricity usage. Partnering principal component analysis with text reports, as explored by de Zarza et al [22], enhances predictive accuracy for forecasting weather and traffic volume.

The next step in the evolution of LLMs for health diagnostics involves fusing diverse information sources. Girdhar et al [23] aligned video, text, and audio by creating unified image embeddings. Xu et al [24] paired images with radiology reports. While these prior efforts illustrate the potential of LLMs to synthesize information across modalities, Cascella et al [25] caution that LLMs still face challenges in aligning personal and general information sources effectively.

This paper aims to contribute to the evolving landscape by exploring the use of LLMs to create a human digital twin from diverse information sources. First, we consider a novel integration of digital behavior markers into the language model that are collected from continuous sensor data. Second, we enhance the language model for cognitive health domains by incorporating self-report, traditional clinical assessment, and automated performance scores. Third, we investigate whether LLMs can offer an effective mechanism for creating a digital twin from these varied components that enhances the accuracy of cognitive health diagnosis and the explainability of system inferences.

## Methods

### HDTwin LLM

**Figure 1:**
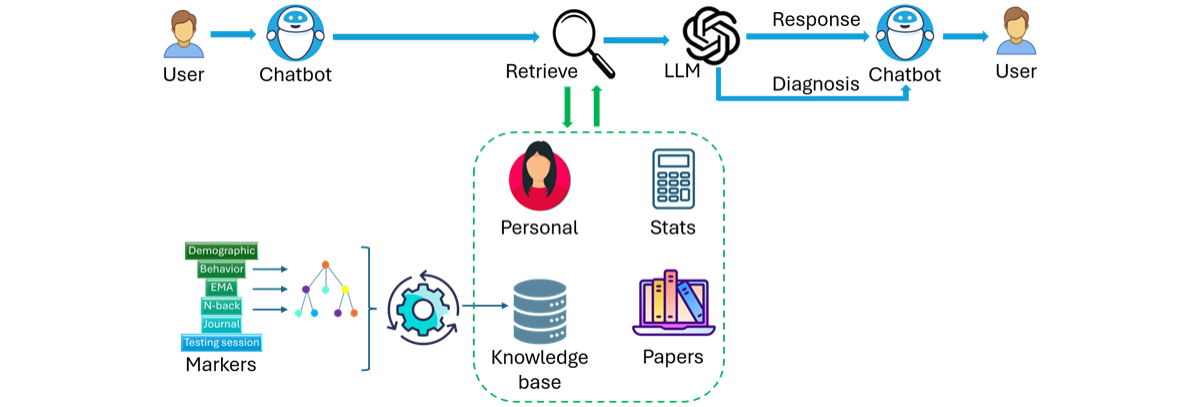
HDTwin information processing pipeline. A user interacts with the LLM interface to request summary information about a person or a suggested diagnosis. Based on the query, HDTwin retrieves personalized markers together with paper abstracts and data from a knowledge base that informs a response. The query response is presented to the user, supporting an ongoing conversation about the person or explanation of the query response. LLM: large language model.

We built HDTwin using LangChain and OpenAI’s GPT-3.5-turbo-0125 language model. As with other GPTs, the model is based on a transformer architecture and uses a self-attention mechanism to aid in capturing dependencies and context within the text. HDTwin’s processing pipeline is illustrated in Figure 1. Using custom LangChain tools, HDTwin retrieves information from personal data, statistical summaries, paper abstracts, and a knowledge base to input as prompts to an LLM, which then generates output for the user in response to a query or diagnosis request. The knowledge base incorporates diverse personal markers which are prompt engineered for input to the language model.

We validate HDTwin using data from 3 studies. Participants in these studies were 124 independent-living older adults, age mean 70.48 (8.72) years, 71.78% female (n=89). Participant recruitment and screening were similar across the studies. Recruitment included community health and wellness fairs, TrialMatch, advertisements on social media, physician referrals, and web-based posts. Inclusion criteria were individuals aged 50 years and older and the ability to speak English; exclusion criteria included current psychoactive substance use; significant auditory visual, or cognitive impairment; presence of a psychiatric, neurologic, or medical condition that greatly attributed to cognitive complaints; and Telephone Interview for Cognitive Status [26] score<26. Participants provided informed consent, and the studies were approved by the Washington State University institutional review board. The source code for HDTwin and a video demonstration of the chatbot interface are available publicly available [27,28].

Each participant was assigned a fictitious name, sampled from a repository [29]. This step was performed to anonymize references to the names that appeared in processed text. Participants were categorized as cognitively healthy older adults (n=75, 60.5%) or older adults with MCI (n=49, 39.5%). To perform these categorizations, at study baseline interviews were conducted, questionnaires were completed (eg, Patient-Reported Outcomes Measurement Information System) [30], and standardized neuropsychological tests evaluating the cognitive domains of memory, language, executive functioning, and attention (3 scores per domain) were administered. These included the Wechsler Adult Intelligence Scale—Fourth Edition [31], Digit Span Forward and Backward subtests, the Delis-Kaplan Executive Function System [32], Category Switching test, the Five Point Test [33], the California Verbal Learning Test [34], and self-reported measures from the Patient-Reported Outcomes Measurement Information System [30]. Jak et al criteria [35] were followed to classify individuals as MCI. These participants were primarily single domain (n=99, 80%), and most met the criteria for amnestic MCI (n=97, 78%).

### Ethical Considerations

These studies were reviewed and approved by the institutional review board at Washington State University. To participate in any of the studies, participants needed to sign an informed consent; each person received compensation between US $55 and US $120 for their participation, consistent with the time demands of the study. All data were anonymized before performing analyses.

### Data

Participant data stem from multiple numeric and text-based marker sources. Language models currently struggle with numeric reasoning for real-valued variables. HDTwin includes agents to summarize and learn models from raw numeric data. To handle the cases where prompts are fed directly to the LLM, however, we transform each real-valued marker to a 0-10 integer scale.

### Demographic Markers

The age, sex, and number of education years of each participant were included as markers.

### Behavior Markers

**Figure 2:**
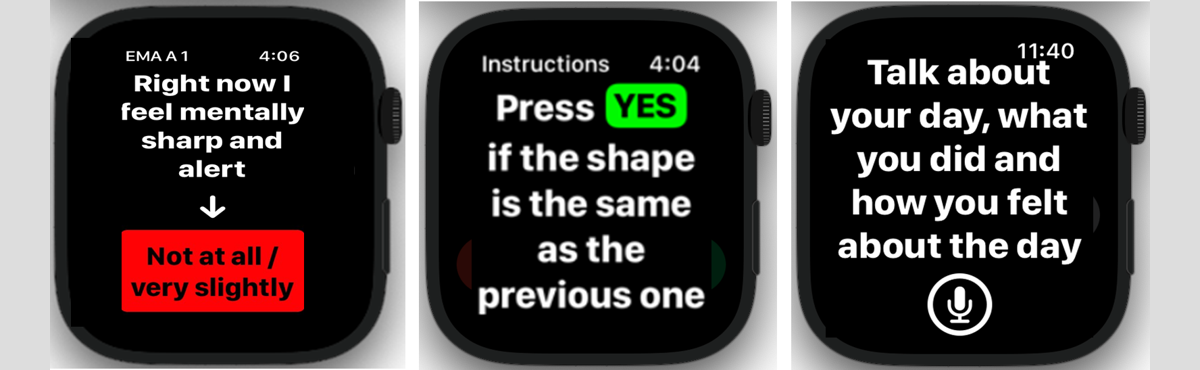
In addition to collecting sensor data, the smartwatch app queries the user for their current state, includes an n-back shape test, and collects daily audio data.

All participants wore a smartwatch (Apple Watch) daily for a minimum of 2 weeks. The watches continuously collected acceleration, rotation, and location data at 10 Hz. From the location coordinates, we defined the participant’s home as the most frequently visited location among the first 300 readings each day. From these data, we extracted activity level (estimated as total acceleration) and distance from home. These values were aggregated by day, and then we calculated the mean and variance over the entire data collection period. The missing data rate was 14%, and missing entries were not included in the calculations. Figure 2 shows screenshots of the smartwatch app.

### EMA Markers

EMA responses were collected 4 times/day at random points within specified time windows. During this session, each participant responded to the prompt “Right now I feel mentally sharp” on a Likert scale of 1=“Not at all” to 5=“Extremely.” For each person, we extract the mean and variance for the EMA response value and response or compliance rate.

### n-Back Markers

Prior research indicated that the *n*-back task, delivered via a tablet, can capture cognitive capacity for older adults as it is influenced by fatigue, mental sharpness, and the environment [9]. We adapted this *n*-back shape test to the smartwatch. The 3 shapes (circle, square, and diamond) were displayed on the watch screen, and participants indicated whether the current shape was the same as the prior. We computed accuracy for each 45-second task.

Earlier studies reveal the importance of considering *n*-back performance in terms of the learning phase (when scores start low but increase sharply) as well as characteristics of performance over the entire sampled period. After the learning phase, daily performance varies with fatigue, mood, mental sharpness, cognitive changes, and environmental factors [36,37]. Applying linear regression to the sequence of daily scores, we extracted the slope for the first 6 scores (the learning rate). We also computed the overall score mean and SD.

### Speech Markers

Each day, participants provided a verbal description of their day in response to the prompt, “Talk about your day, what you did, and how you felt.” Participant responses were collected by the smartwatch. The audio files were then converted to text and fed verbatim to the language models. A total of 2995 audio files were provided by 85 participants across the 3 studies. All descriptions from a single participant were aggregated into 1 text entry per person.

### Testing Session Markers

#### Overview

From the administered cognitive assessment tests that were delivered orally at the study baseline, we converted the recorded testing session audio files to text. Based on the experimenter script, we input experimenter questions as prompts to the LLM. The purpose of this step was to have the language model quantitatively evaluate participant responses based solely on the recorded interaction. This contrasts with the traditional method of using a predefined set of rules to determine the score for each assessment task. There were 23 types of participant responses, generated from tests for phonemic and semantic fluency, list learning and delayed memory, cognitive screening, and health literacy.

As shown in Table 1, we asked the LLM to extract and rate participant responses on a scale of 1=poor to 5=excellent or report as missing. While some prompts included a list of correct answers (eg, “Where is our lab located?”), many did not (eg, “list all of the animals you can think of”). In the second case, response rating consequently relied on the model’s reasoning. Of the 124 baseline testing session transcripts, 116 had at least 3 responses and were input to the LLM.

**Table 1:** Example prompt for processing baseline testing session data. The user provides the system message and prompt; HDTwin generates the corresponding response.

	Example user system message and prompt with corresponding HDTwin response
System message	You are a helpful assistant that extracts and rates participant responses to interview questions.
Example prompt	The following is an unlabeled transcript of an interview between an interviewer and a participant: “all right so the recordings on again for the next task I'm going to.” There should be a part of the transcript where the interviewer asks the participant to list as many words that start with the letter S as they can think of. Using only exact quotes from the transcript, provide the participant’s response to the interviewer’s question. Then, using a scale of 1=poor to 5=excellent, rate the participant’s ability to think of words that start with S. Briefly explain your rating. If you cannot find the participant’s response, respond only with: could not find the participant’s response.
Example response	Participant’s answer: Sun, stairs, startled, stars, stilt, stern, south, song. Rating: 5. Explanation: The participant was able to list multiple words that start with the letter S, demonstrating a strong ability to recall words.

**Figure 3:**
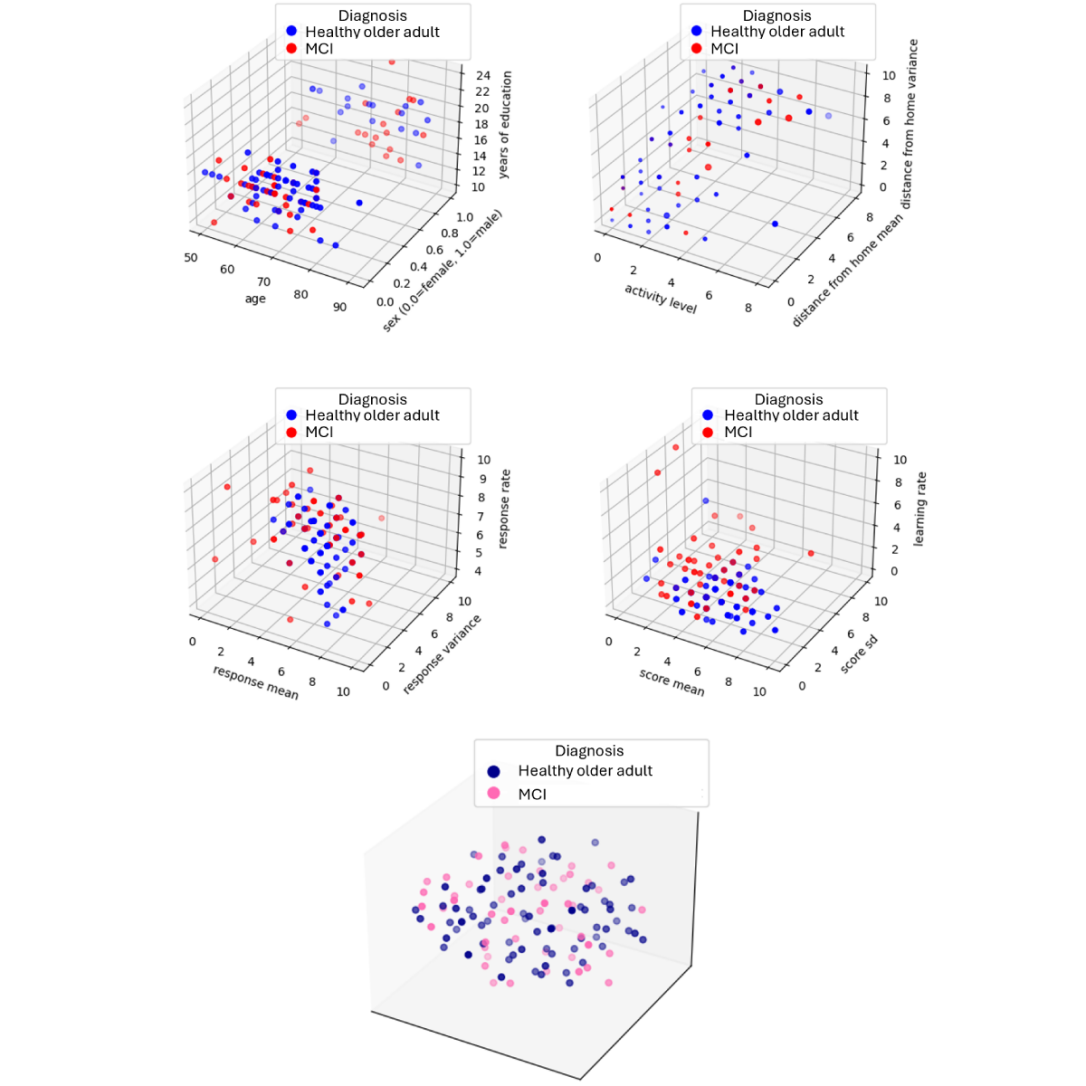
Distribution of healthy participants and those with MCI based on HDTwin markers that include (from upper left): demographics, behavior, EMA response, and n-back scores. The bottom graph shows a t-sne plot of all quantifiable features. Text input from journals and testing sessions are not included in the plots. EMA: ecological momentary assessment; MCI: mild cognitive impairment.

Figure 3 illustrates the distribution of participants between the healthy and MCI categories based on each type of quantifiable marker (ie, demographics, behavior, EMA response, and *n*-back score). As can be seen from the plots, the categories are highly intermixed for each category of marker as well as the t-sne combination of these markers. The high degree of intermixing between categories indicates that there are no clear, simple class boundaries separating the groups, making diagnosis prediction more complex. This complexity necessitates leveraging all available information to improve prediction accuracy, which requires integrating information with diverse modalities, granularities, and availability. This challenge motivates the adoption of an LLM-based method for this task, which is well-suited to handling and synthesizing such heterogeneous data.

#### Knowledge Base Construction

To predict cognitive diagnoses with LLMs, we constructed a prompt template that combines two primary sources of context to be combined by the LLM: (1) insights gleaned from external knowledge about the field, and (2) insights gathered from personalized data markers.

We use these information sources by transforming them into text prompts for the LLM. To integrate insights from the field, each prompt is framed based on a finding from a relevant publication. Language models have demonstrated their ability to summarize medical research papers [38]. In contrast, we use such research papers as just 1 piece of the digital twin to provide more comprehensive reasoning about a person’s state. In this context, we introduce a novel use of such articles, leveraging them as sources of external neuropsychology insights. Textbox 1 provides a summary of the relevant literature sources we included for HDTwin.

**Rules found in external information sources. These rules represent summaries of statements found in the included research papers.**

- More hours spent outside the home and greater distance from home were associated with better cognitive function [6].
- Physical exercise demonstrated a protective factor for mild cognitive impairment (MCI) and was associated with higher mean semantic memory and executive function [8,39].
- Association was found between participant sentence complexity and levels of cognitive impairment [2].
- Individuals with MCI were observed to perform worse on shape tests than healthy older adults [4].
- Response or compliance rate was greater for healthy older adults compared to mild cognitive impairment [5].
- Detected changes in behavior patterns were early indicators of cognitive decline [40].

Three types of LLM prompts are created. First, prompts that process text directly (ie, journal entries and testing session transcripts) are formalized based on a statement found in the corresponding research paper (ie, external knowledge). HDTwin contains 11 of these rules, such as “if journal_text has a small vocabulary, short sentences, and/or low sentence complexity then more likely mild cognitive impairment” [41]. The full set of rules is provided in Table 2. The LLM must interpret the meaning of the rule in the context of the input text. Second, as described earlier, the LLM is directed to generate numeric ratings of the testing session participant responses.

**Table 2:** HDTwin text processing rules. These results are used to process information found in text including journal entries and interview assessments.

Marker set	Rule
Speech	If journal_text is empty the more likely mild cognitive impairment If journal_text has a large vocabulary, long sentences, and/or high sentence complexity then more likely healthy If journal_text has a small vocabulary, short sentences, and/or low sentence complexity then more likely mild cognitive impairment If journal_text uses positive emotion words then more likely healthy If journal_text uses negative emotion words then more likely mild cognitive impairment If journal_text appears to have more than 1 entry then more likely healthy If journal_text appears to only have 1 entry then more likely mild cognitive impairment
Baseline testing session	if some interview_assessment ratings are <= 3 then more likely mild cognitive impairment if most interview_assessment ratings are >= 4 then more likely healthy if the interview_assessment explanations suggest the participant confidently answered the questions correctly then more likely healthy if the interview_assessment explanations suggest the participant struggled to answer the questions correctly then more likely mild cognitive impairment

Third, a decision tree algorithm processes numeric markers from the training set, learning a concept distinguishing healthy older adults from MCI. The purpose of these models is to provide a mechanism for learning from real-valued numeric data and demonstrate the ability of HDTwin to harness heterogeneous types of information. Decision trees are advantageous because the learned model is easily interpretable. In particular, these trees are automatically converted to if-then rules that are provided to the LLM in a prompt. From the large set of possible rules, we include those that support ≥10% of the training data and the probability of rule occurrence in the tree is >70%. In the case of single-term rules resulting from decision stumps, the rule inversion is also added. This process resulted in 20 rules, such as “if distance_traveled_from_home > 4.5 then more likely healthy” and “if physical_activity_level ≤ 1.5 and EMA_compliance ≤ 9.5 then more likely mild cognitive impairment.” The full set of decision tree-generated rules is provided in Table 3.

**Table 3:** HDTwin numeric processing rules. These results are created from the trained decision trees and are listed with the corresponding Pr^a^ and Pa^b^.

Rule	Pr	Pa
If distance_traveled_from_home > 4.5 then more likely healthy	100.00	6
If distance_traveled_variance > 8.0 and physical_activity_level ≤ 5.5 then more likely healthy	91.67	12
If physical_activity_level ≤ 1.5 and EMA_compliance ≤ 9.5 then more likely mild cognitive impairment	85.71	7
If mental_sharpness_variance > 2.5 and mental_sharpness_mean > 4.5 then more likely mild cognitive impairment	83.33	6
If shape_score_sd ≤ 2.5 and mental_sharpness_variance ≤ 2.5 then more likely healthy	81.82	22
If distance_traveled_variance > 8.0 then more likely healthy	80.00	15
If mental_sharpness_variance ≤ 2.5 and mental_sharpness_mean ≤ 4.5 then more likely mild cognitive impairment	80.00	5
If mental_sharpness_mean > 5.5 and physical_activity_variance ≤ 8.0 then more likely healthy	79.17	24
If sex = male and age ≤ 79.5 then more likely mild cognitive impairment	77.78	9
If mental_sharpness_variance > 2.5 then more likely mild cognitive impairment	77.78	9
If sex = female and shape_learning_rate ≤ 4.5 then more likely healthy	76.47	17
If shape_score_sd > 2.5 and shape_learning_rate > 1.5 then more likely mild cognitive impairment	75.00	16
If physical_activity_variance ≤ 0.5 then more likely mild cognitive impairment	75.00	8
If mental_sharpness_mean ≤ 4.5 then more likely mild cognitive impairment	75.00	8
If shape_score_sd > 2.5 then more likely mild cognitive impairment	70.59	17
If shape_score_sd ≤ 2.5 then more likely healthy	60.61	33
If distance_traveled_variance ≤ 8.0 then more likely mild cognitive impairment	60.61	33
If physical_activity_variance > 0.5 then more likely healthy	57.50	40
If mental_sharpness_variance ≤ 2.5 then more likely healthy	57.50	40
If mental_sharpness_mean > 4.5 then more likely healthy	56.10	41

^a^Pr: probability of the rule occurring in the decision tree.

^b^Pa: number of participants supporting the rule.

#### Chatbot Agent Tools

The chatbot agent is designed as a Python LangChain OpenAI tools agent. We created custom tools that allow the chatbot agent to plan and take actions to solve specialized tasks. By integrating these tools, HDTwin links the LLM with additional sources of information and additional functionality, yielding a more complete digital twin. These agent tools include the following.

- Participant data retriever tool: We embedded and stored the test dataset in a Facebook AI Similarity Search vector database. We then created a LangChain retriever tool for the database that the agent can query to find markers for a participant.
- Reference data calculation tool: We wrapped a LangChain pandas agent in a custom tool that loads the reference data into a pandas DataFrame. The agent can query this calculation tool to get on-the-fly summary statistics for participants, such as grouping data by diagnosis category and then calculating the mean for a marker of interest. This tool allows HDTwin to interactively answer questions regarding a particular person, including their scores on each assessment component and how the scores compare with the participant cohort.
- Knowledge base retriever tool: We embedded and stored the knowledge base in a Facebook AI Similarity Search vector database. Similar to the participant data retriever tool, the agent can query a LangChain retriever tool to find knowledge in the database that may interest the user. For example, HDTwin may quote one of the findings from the literature as part of the reason it predicted a particular diagnosis category for a participant.
- Paper abstract retriever tool: Using the LangChain PubMed app programming interface, we created a tool that searches PubMed abstracts for information that may be relevant to the query tasks. Text from the abstracts is included verbatim as input. The tool can also be leveraged to include literature from other public domain sources.
- Diagnosis classification tool: Using the same classification prompting strategy described in an earlier section, we created a LangChain structured tool that uses a custom chat chain to classify a participant as a healthy older adult or MCI. The chain constructs a prompt using information from the knowledge base and personalized markers to request that the language model generate predictions (see the Diagnosis Prediction section for more details about diagnosis prompt construction).

**Figure 4:**
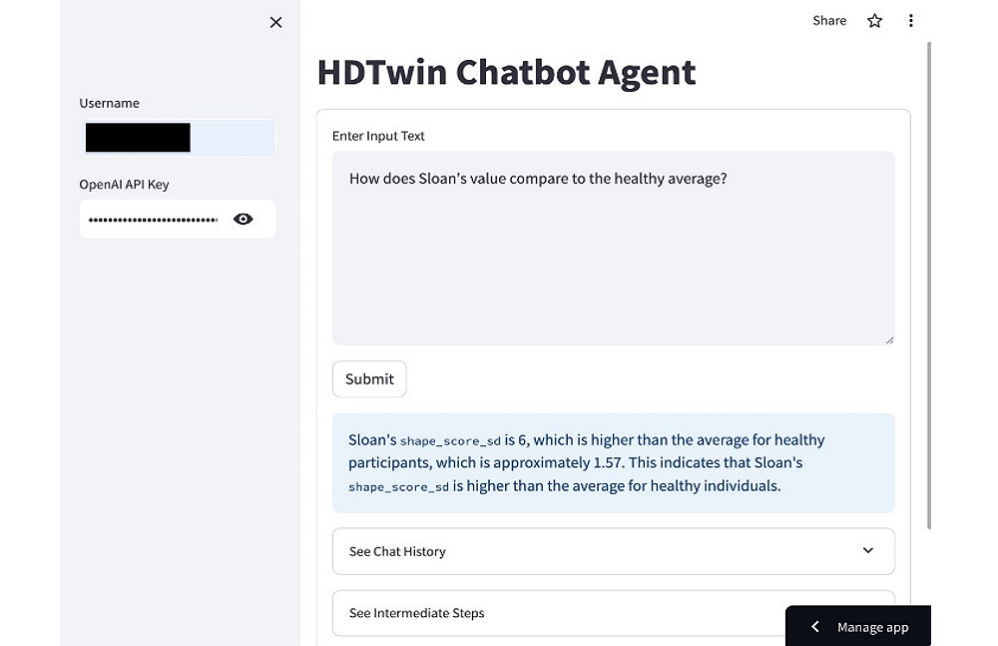
The HDTwin chatbot interface with an example prompt and response for a query regarding one of a person’s n-back score statistics. Users can see the agent’s message memory using the “Chat History” dropdown and the agent’s planning and execution steps using the “See Intermediate Steps” dropdown. A video demonstration of the chatbot is available on the web [28].

We designed the agent with an interface built using the Python (Python Software Foundation) *streamlit* library. Figure 4 [28] provides a screenshot of this interface. As the figure demonstrates, HDTwin leverages the participant’s personal markers (demographics, behavior, EMA response, *n*-back scores, and testing session data), rules generated from the training cohort, and literature from the field to respond to user queries. Upon request, the agent lists the steps that were performed by generating a response, pulling from the message memory as needed (ie, interaction history), as well as the information sources.

### Diagnosis Prediction

#### Overview

The long-term goal of HDTwin is to build a digital twin representing the cognitive health state of a physical human. The digital twin can respond to queries about the person’s behavior, task performance, and predicted cognitive health diagnosis. Unlike traditional machine learning tools, the system will interactively explain its reasoning and ingest additional information that is provided on the fly.

In this paper, we consider the role of HDTwin in performing cognitive diagnosis prediction. Specifically, we seek to validate the ability of HDTwin to perform cognitive diagnosis from a fusion of heterogeneous information. Using the LLM model, we constructed a classification pipeline that we performed for each participant in the test set. The pipeline begins by constructing the context needed to form a prompt. Textbox 2 provides an example of this context, which primes the language model to perform reasoning and generate a response. As shown in the example, the prompt includes instructions, the knowledge base rules that it references, and participant markers. The LLM is prompted to return a response with the diagnosis, which we extract using a regular expression pattern search. Upon request, the LLM will also provide an explanation for the diagnosis. By default, HDTwin provides a short (ie, few-sentence) explanation. The full explanation can be extracted upon request. The latter not only provides insight into the LLM’s reasoning but can also help with response accuracy using chain-of-thought reasoning [42].

**Example cognitive diagnosis prompt. The user inputs the system message and prompt, and HDTwin generates the corresponding response. In this case, the classification of diagnosis from HDTwin is correct.**

**Example user system message and prompt with corresponding HDTwin response**

**System message:**

You are a knowledgeable health assistant designed to diagnose someone as “healthy” or “with mild cognitive impairment” using diagnosis rules and relevant personal information.

**Example prompt:**

Diagnose Sloan as healthy or mild cognitive impairment by applying the following rules to Sloan's information.

Diagnosis Rules:

Rule #1: if shape_score_sd > 2.5 then more likely mild cognitive impairment

Rule #2: if shape_score_sd <= 2.5 then more likely healthy

Rule #3: if shape_score_sd > 2.5 and shape_learning_rate > 1.5 then more likely mild cognitive impairment

Rule #4: if journal_text has a large vocabulary, long sentences, and/or high sentence complexity then more likely healthy

Rule #5: if some interview_assessment ratings are <= 3 then more likely mild cognitive impairment

Sloan's Information:

shape_learning_rate: 9

shape_score_sd: 6

journal_text: made a list and went to the store and got everything on it and didn't alter from it

interview_assessment: Category Fluency (Animals): Participant's Answer: “turkey”

Rating: 2

<text removed for brevity>

Answer using the format:

Diagnosis: ...

Explanation: ...

**Example response:**

Diagnosis: Mild cognitive impairment

Explanation: Based on the provided information, Sloan’s shape_score_sd is 6, which is greater than 2.5, and the shape_learning_rate is 9, indicating a high learning rate. According to Rule #3, when shape_score_sd is greater than 2.5 and shape_learning_rate is higher than 1.5, the individual is more likely to have mild cognitive impairment. Additionally, some of Sloan’s interview_assessment ratings are <= 3, such as the ratings for category fluency (animals) and letter fluency (F), further supporting the diagnosis of mild cognitive impairment.

We measure prediction performance using accuracy, MCI precision and recall, and the Matthews correlation coefficient. To provide a baseline for comparison, we trained 3 traditional classifiers using numeric markers. We trained the models on the same participants used to build the LLM knowledge base and evaluated performance using the same test set. The baseline classifiers are random forest, gradient boosting, and XGBoost.

#### Reference, Training, and Test Sets

Traditional supervised machine learning uses data from a training set to train a model. In contrast, HDTwin performs retrieval-augmented generation by combining information retrieval with a generative model [43]. In HDTwin, therefore, training data are used to build a knowledge base of text, data, and rule prompts for the language model to retrieve. Following literature guidelines for evaluating LLMs [44], we created 3 sets—reference, training, and test—from the original participant group (N=124). Data from the reference set support the chatbot functions. A subset of the reference group is selected as the training set. In our experiments, the training set is used to build a knowledge base of text input, numeric data, and decision tree rules for HDTwin to perform diagnosis prediction. Participants not in the reference (and training) set are reserved to form a test set. Prediction performance is determined based on these held-out data.

Participants included in the training and test sets were required to have complete behavior, EMA, and *n*-back markers, as these markers were essential for conducting an ablation analysis of their contribution to diagnosis prediction. To ensure a uniform class distribution, 24 participants were randomly selected from each diagnosis group (MCI and healthy older adult) to form a test set of 48 participants. Of the 76 reference set participants, 51 were labeled healthy (67.1%) and 25 were labeled MCI (32.9%). For the training set, we randomly selected 25 reference set participants from each category (51 labeled healthy and 25 labeled MCI), resulting in an equal split.

## Results

Table 4 demonstrates an interaction with the HDTwin chatbot agent in which the agent executes custom tools and accesses a memory cache for conversation history. The generated rules were used to form two classification prompt variants, one with a requested explanation that aids in understanding the LLM’s decision-making process. Experiment results showed no substantial difference between these variations, so results are reported for the smaller, diagnosis-only prompt. Lastly, HDTwin performs a wrapper-based selection of rules to include in the diagnoses. In the wrapper method, a greedy search is used to identify the final set of features, or rules, to include [45].

**Table 4:** Example chatbot interaction. User-input text is highlighted with bold font. Each HDTwin response is listed with the chatbot agent tools that were used to generate the corresponding response.

Prompt		Tools Used
**What do you know about Sloan?**		
	Sloan is a 57-year-old female with 14 years of education. She has a shape score SD of 6 and a shape learning rate of 9. Sloan’s EMA^a^ compliance is rated at 8.0, and her physical activity level is 4.0. She has a mental sharpness mean of 6.0 and a mental sharpness variance of 5.0. Sloan’s journal entry mentions activities such as making a list, going grocery shopping, using the EMMA app to remind her to take pills, and engaging in social and leisure activities. If you would like more specific information about Sloan or her cognitive health status, please let me know.	Participant data retriever
**Would you diagnose Sloan as mild cognitive impairment?**		
	Sloan has been diagnosed with mild cognitive impairment based on her cognitive assessment results. If you have any further questions or need more information, feel free to ask.	Diagnosis
**Can you explain why you made that diagnosis?**		
	Sloan has been diagnosed with mild cognitive impairment based on her cognitive assessment results. The diagnosis is supported by the fact that Sloan’s shape_score_sd is greater than 2.5 and her shape_learning_rate is greater than 1.5, meeting the criteria for mild cognitive impairment.	Message memory and diagnosis
**How does Sloan’s shape_score_sd compare to the average?**		
	Sloan’s shape_score_sd value is 6, which is higher than the average value of approximately 1.57 for healthy participants. This indicates that Sloan's shape_score_sd value is significantly higher compared to the average value of healthy participants.	Message memory and training calculation

^a^EMA: ecological momentary assessment.

Table 5 summarizes the diagnostic accuracy of HDTwin compared to baseline classifiers. These include results for the LLM using wrapper selection, a single type of information source, or all available rules. Due to LLM nondeterminism, each variation was executed 30 times; the mean and SD were reported. To quantify the improvement gained by the wrapper approach, we computed an unpaired 1-tailed *t* test, comparing the LLM wrapper results against the best single marker set (*n*-back; *P*<.001) and the best traditional classifier (XGBoost; *P*<.001).

**Table 5:** Prediction performance. Performance is reported in terms of prediction accuracy, precision, and recall for the MCI^a^ category, and MCC^b^.

Classification			Accuracy		MCI precision		MCI recall		MCC
**LLM**									
	Demographics	0.48±0.00		0.47±0.00		0.33±0.00		–0.04±0.00	
	Behavior	0.43±0.02		0.43±0.02		0.43±0.04		–0.14±0.05	
	*n*-back	0.75±0.00		0.93±0.00		0.54±0.00		0.55±0.00	
	EMA^c^	0.65±0.00		0.89±0.00		0.33±0.00		0.37±0.00	
	Journal	0.57±0.04		0.56±0.03		0.63±0.05		0.13 ± 0.07	
	Test session	0.61±0.01		0.78±0.01		0.30±0.02		0.28±0.02	
	All	0.56±0.01		0.64±0.02		0.29±0.02		0.15±0.02	
	Wrapper	*0.77±0.02* ^d^		*0.88±0.05*		*0.63±0.03*		*0.57± 0.05*	
Random forest			0.62±0.04		0.86±0.03		0.27±0.00		0.31±0.07
Gradient boosting			0.65±0.01		0.89±0.01		0.35±0.03		0.39± 0.02
XGBoost			0.67±0.00		0.83±0.00		0.42±0.00		0.38±0.00

^a^MCI: mild cognitive impairment.

^b^MCC: Matthews correlation coefficient.

^c^EMA: ecological momentary assessment.

^d^Italicized values indicate the best performer for each metric.

## Discussion

### Principal Results

The goal of this study was to explore the use of LLMs as a mechanism to fuse multimodal information relevant to understanding and predicting the cognitive health diagnosis for an individual. As demonstrated in Table 4, the HDTwin chatbot agent conversationally provides answers related to the dataset and cognitive diagnosis. When prompted for a diagnosis for a particular participant, it succinctly responds with a predicted class label. When asked to explain the diagnosis, the agent correctly cites a rule from the knowledge base, though the chatbot may offer only a subset of rules that were used for the inference.

We note that when the chatbot is requested to compare a participant to the training set, the LLM calls the training calculation tool, which generates and executes the code to select, filter, and summarize the underlying DataFrame (eg, generating {'query': “df[df['diagnosis'] == 'healthy'] ['shape_score_sd'].mean()”}). In this case, the training calculation tool produces the correct value (1.57). Because LLMs are nondeterministic systems, the chatbot is not guaranteed to return the same response each time.

Nondeterministic behavior also affected the classification results. This behavior is evidenced by the nonzero performance SDs that are listed in Table 5. We further observed that longer prompts (eg, journal and testing sessions) generally led to less consistent performance (eg, higher SD in Table 5). As the prompt text increases in length, there is a higher risk of divergence due to the model latching onto different parts of the prompt in different ways across each run, creating more variability in the output.

Another factor influencing classification performance is the type of information that is used and the way the information is incorporated into the LLM prompt. While HDTwin can use all information sources, Table 5 illustrates that not all information was equally effective at discriminating between diagnosis classes. Of the 6 marker sets, *n*-back offers the most predictive rules (0.75 accuracy). While the wrapper method occasionally selected the text-based journal and testing session markers, this marker set did not perform the best. The top-performing case, with 0.81 accuracy, was a run with the combination of *n*-back and behavior markers. The worst-performing cases combined demographic markers with behavior markers. This is not surprising considering only 2 rules using demographic markers were supported with a high probability of occurrence in the training set. Our results show that the LLM wrapper method significantly outperformed the best traditional classifier as well as the best individual marker set (*n*-back). Results for the wrapper method are comparable to reported results, which use MRI and cerebrospinal fluid to perform a similar task, yielding an accuracy of 76.4% [46]. These findings provide evidence supporting in-the-home data collection and the design of LLM technologies for improved MCI diagnosis.

### Limitations

This study faced several limitations, including missing data across several of the marker sets, reliance on an LLM to extract and label participant responses to testing session questions, and a small sample size of training participants (n=50) whose data formed the knowledge base. Additionally, LLM nondeterminism affects the reproducibility of the results. In the future, we plan to explore other LLMs for the HDTwin chatbot agent and diagnosis classification task, as well as improve prediction accuracy with more in-depth prompt engineering and an expansion of the knowledge base.

Many of HDTwin’s data sources are continually updated. These include the behavior markers, *n*-back scores, and journal entries. The rules can be periodically updated, allowing HDTwin to adapt to this new information dynamically. Our approach does not finetune the model but provides new context. Future work will investigate how to direct the LLM to model the historical evolution of the prompts and forecast the future state of the individual. In this setting, the digital twin can provide a tool for testing scenarios and predicting outcomes of various behavior changes and other types of changes. Such a tool can help optimize potential treatment decisions for each person before they are administered.

### Conclusions

In this paper, we built a custom agent called HDTwin for interactively exploring a multimodal, in-the-wild health dataset (N=124), supporting the creation of a digital twin. The HDTwin digital twin contains an agent that supports the diagnosis of MCI, guiding more informed and actionable cognitive assessment. To build this classifier with an LLM, we explored the predictive capability of diverse individual and fused health markers. A fusion of knowledge and participant data from different marker sets yielded the strongest performance. Our findings indicate that HDTwin significantly outperforms traditional classifiers in diagnosing MCI. Integrating diverse data sources through LLMs provides a comprehensive view of cognitive health, enhancing diagnosis and intervention strategies. Future studies can continue to explore approaches for increasing the accuracy of LLMs to improve the accuracy of custom agents like HDTwin for aiding clinicians with health care diagnosis and prediction of outcomes.

## Data Availability

The datasets analyzed for this study are available from the authors upon reasonable request. The code for HDTwin and the smartwatch *n*-back task are available on the web [27,47].

## Abbreviations

EMA: ecological momentary assessment
LLM: large language model
MCI: mild cognitive impairment
